# Needle tracking in low-resolution ultrasound volumes using deep learning

**DOI:** 10.1007/s11548-024-03234-8

**Published:** 2024-07-13

**Authors:** Sarah Grube, Sarah Latus, Finn Behrendt, Oleksandra Riabova, Maximilian Neidhardt, Alexander Schlaefer

**Affiliations:** Institute of Medical Technology and Intelligent Systems, Hamburg University of Technology, Hamburg, Germany

**Keywords:** Volumetric ultrasound imaging, Deep learning, Needle tip detection, Real-time, Sparse feature learning

## Abstract

**Purpose:**

Clinical needle insertion into tissue, commonly assisted by 2D ultrasound imaging for real-time navigation, faces the challenge of precise needle and probe alignment to reduce out-of-plane movement. Recent studies investigate 3D ultrasound imaging together with deep learning to overcome this problem, focusing on acquiring high-resolution images to create optimal conditions for needle tip detection. However, high-resolution also requires a lot of time for image acquisition and processing, which limits the real-time capability. Therefore, we aim to maximize the US volume rate with the trade-off of low image resolution. We propose a deep learning approach to directly extract the 3D needle tip position from sparsely sampled US volumes.

**Methods:**

We design an experimental setup with a robot inserting a needle into water and chicken liver tissue. In contrast to manual annotation, we assess the needle tip position from the known robot pose. During insertion, we acquire a large data set of low-resolution volumes using a 16 $\times$ 16 element matrix transducer with a volume rate of 4 Hz. We compare the performance of our deep learning approach with conventional needle segmentation.

**Results:**

Our experiments in water and liver show that deep learning outperforms the conventional approach while achieving sub-millimeter accuracy. We achieve mean position errors of 0.54 mm in water and 1.54 mm in liver for deep learning.

**Conclusion:**

Our study underlines the strengths of deep learning to predict the 3D needle positions from low-resolution ultrasound volumes. This is an important milestone for real-time needle navigation, simplifying the alignment of needle and ultrasound probe and enabling a 3D motion analysis.

## Introduction

In various clinical interventions, accurate needle placement is crucial for optimal diagnosis and treatment results, e.g., during biopsies or epidural punctures. Two-dimensional (2D) ultrasound (US) imaging provides real-time visualization of the needle and the punctured tissue and thus optimized positioning of the needle. However, one common problem when tracking the needle in 2D is the need for precise alignment of the needle with the US probe. Movements of needle and tissue outside the imaging plane cannot be visualized. In practice, the needle axis often deviates from this ideal alignment, which leads to less accurate needle tip detection and longer interventions depending on the experience of the physician. Therefore, recent studies have investigated needle tip detection in three-dimensional (3D) US images [1, 5, 11, 15]. However, extracting the needle tip position from 3D US images still faces several challenges. For example, imaging artifacts caused by acoustic impedance differences at the needle are amplified by the increased number of sound waves that are emitted in different spatial directions. Analytical methods for 3D needle tip detection already show robust results but are time-consuming and less suited for real-time applications [15]. Recent studies have investigated deep learning approaches for 3D needle tracking and showed promising results [9, 12, 13]. In these approaches, an initial needle segmentation is followed by the determination of the needle tip position. Alternatively, semantic voxel-wise segmentation methods have been proposed [10]. The analyzed US volumes have been composed of high-resolution 2D images from a manually rotated probe or a motorized 3D probe [3, 14]. However, acquiring, annotating, and analyzing these large and high-resolution US volumes is very time-consuming, impeding real-time application. Even though deep learning approaches enable fast image processing of less than 0.2 s [14], the proposed manual annotation of 3D volumes is highly observer-dependent and requires high image quality.

Summarizing the current literature, deep learning approaches seem to be a promising approach in order to enable real-time needle tip tracking in volumetric ultrasound images. However, until now, high-resolution focused B-Mode ultrasound volumes have been used for this, limiting the real-time capability by mainly two factors: first the low acquisition rate of 1.6–1.8 Hz [3] and second the large data size of one US volume [10]. The latter is an important factor that strongly influences the time required for subsequent image processing steps. Hence, in order to enable real-time needle tracking, both, data acquisition and processing have to be improved.

**Fig. 1:**
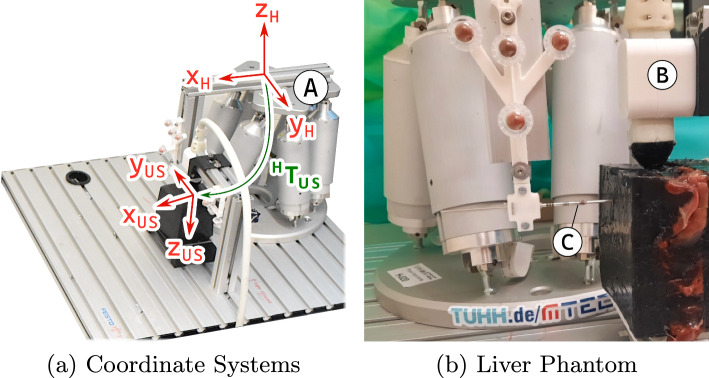
Experimental setup: for data acquisition we use a hexapod robot (A) to drive the needle (C) inside a phantom. A matrix transducer containing 16 $\times$ 16 elements (B) is used for ultrasound needle tracking. On the right is depicted the experimental setup for data acquisition in liver soft-tissue

**Fig. 2:**
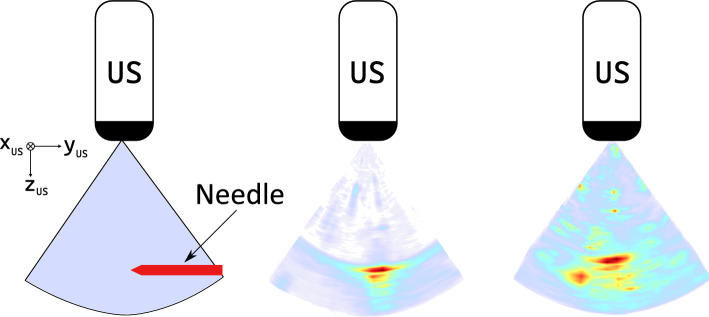
US volume with needle: a schematic drawing (left) of a needle position relative to the ultrasound transducer (US) is shown with the corresponding ultrasound volume in water (middle) and liver (right).The ultrasound volume is visualized using maximum intensity projection

High volumetric imaging rates are feasible with custom US matrix transducers that incorporate the same amount of elements for imaging as channels available in the US system. Hence, time-consuming multiplexing of the ultrasound elements can be avoided. However, the disadvantage associated with these probes, which enable real-time volumetric data acquisition, is the poorer image resolution. Bengs et al. [2] used a 16 $\times$ 16 element matrix transducer to perform real-time soft tissue motion analysis in low-resolution US images using deep learning, which outperformed conventional approaches. However, the feasibility of detecting the needle tip position in low-resolution US volumes using deep learning approaches has not been shown yet. An important prerequisite when using deep learning architectures for needle tip detection is the generation of reliable ground truth. In the current literature, either clinical experts manually detect the needle tip for each volume [14] or conventional analytical algorithms are used. However, both methods are error-prone and time-consuming, making it difficult to investigate and implement accurate deep learning approaches.

As real-time capability is particularly important for needle insertions, our work addresses the problem of 3D needle tip detection in low-resolution US volumes acquired with a 16 $\times$ 16 element matrix probe. We aim to increase the temporal volume acquisition rate with the trade-off of minimized image resolution. We hypothesize that with the application of deep learning methods, we do not need to acquire high-resolution volumes that can be easily annotated by clinical experts. Instead, we design an experimental setup to acquire a large data set with our needle being inserted in water or a tissue phantom in a reproducible fashion. In this way, we acquire data with a known orientation relative to the US volume, which directly serves as ground truth for training. We perform several evaluations using robot positions as training target. We define a deep learning approach that directly extracts the needle tip position from the low-resolution US volume without the need for prior needle segmentation or manual annotation.

## Material and methods

We present an experimental setup for automated data acquisition of needle punctures using a robot. US volumes of needle insertions are acquired in water as an imaging medium as well as in chicken liver tissue. We perform needle insertions parallel to the ultrasound coordinate system and at tilted angles. For needle tip detection, we propose a deep learning approach and compare its performance with a conventional segmentation approach.

### Experimental setup and calibration

An overview of our experimental setup for data acquisition is depicted in Fig. 1. Our setup contains a hexapod robot, a US system with a custom volumetric US probe and a needle. The needle has a trocar needle tip and a diameter of 2.15 mm. The hexapod (Hexapod H-820, PI, DE) with axial repeatability of 20 $\upmu$m drives the needle relative to the volumetric US probe which is rigidly mounted to a base plate. The US probe (Vermon, FR) contains 16 $\times$ 16 elements embedded at a pitch of 0.3 mm and has a central frequency of 3 MHz. Volumetric image data are acquired with a 256-channel US system (Griffin, Cephasonics, USA) by connecting each element to an individual channel without multiplexing.

The transformation matrices and notations of our setup are depicted in Fig. 1a. First, the transformation between hexapod base (H) and needle tip (NT) in the hexapod coordinate system ($^{\mathrm{{H}}}T_{\mathrm{{NT}}}$) is estimated with a hand-eye-calibration and the QR24 algorithm [4]. For needle calibration, we use external markers attached to the needle shaft and a tracking camera (fusionTrack 500, Atracsys, CH) with a resolution of 0.09 mm and a temporal sampling rate of 200 Hz. We report a translational error of 0.07 mm and a rotational error of ${0.07}^{\circ }$ based on 761 different poses of the robot. Second, we estimate the transformation from hexapod (H) to US (US) coordinate system ($^{\mathrm{{H}}}T_{\mathrm{{US}}}$), assuming parallel coordinate axes and a pure translational transformation. We determine the needle tip position in acquired US volumes using conventional image processing methods. The translation offset between the US and hexapod coordinate systems is calculated by minimizing the mean error between the detected needle tip positions in the US volume and the corresponding hexapod poses. Please note that the needle tip ground truth positions in the hexapod coordinate system are given in mm; hence, we divide them by the pixel resolution of 0.3 mm to get the hexapod pose in pixel units.

### Data set acquisition

We use the open-source framework **SUPRA** [6] to acquire focused B-Mode US volumes with a sampling frequency of 4Hz, an imaging depth of 5mm to 40mm, and an opening angle of ${70}^{\circ }$. We apply beamforming and construct our US volumes from 16 beams while assuming a constant speed of sound of 1500 m/s. This results in US volumes of 117 $\times$ 134 $\times$ 134 pixels along the depth and lateral axes, respectively. Assuming a pixel resolution of 0.3 mm in all dimensions we report an effective field of view (FOV) of 5–40mm along the depth axis and a maximum lateral width of 40.35mm. We track the needle movement with a temporal sampling rate of 200 Hz by recording the hexapod positions.

We perform needle insertions in water as well as in chicken liver tissue. We manufacture a phantom with a fresh chicken liver embedded in 10% gelatine concentration. To enhance speckle, we add graphite powder to the gelatine before pouring. The phantom is depicted in Fig. 1b. Exemplary low-resolution US volumes of needle insertions acquired in water and liver are shown in Fig. 2. The needle is inserted in $y_\text {H}$-direction over a distance of 15 mm. During needle insertion, we constantly acquire US volumes and track the needle movement based on the hexapod position.

**Fig. 3:**
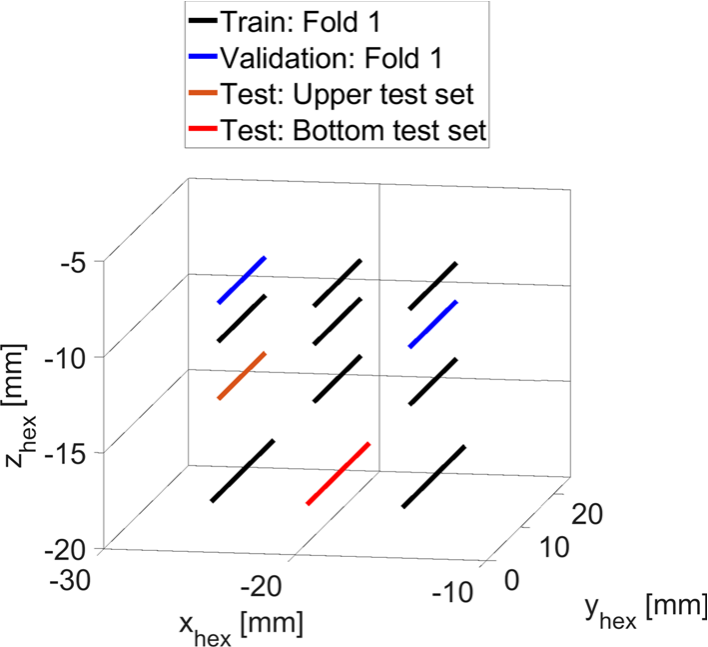
Trajectory showing the acquired needle positions in water and liver in hexapod coordinates. The colors indicate exemplarily for fold 1 whether data are used for training, validation, or testing. Needle insertion is performed along positive $y_{\mathrm{{H}}}$ direction. Note that we can differ between a test set at medium and bottom depth

First, we perform twelve needle insertions parallel to the $y_\text {US}$-direction of the US coordinate system. Based on the orientation of the setup components shown in Fig. 1a, we assume that the needle moves in the negative $y_\text {US}$-direction of the US coordinate system with a velocity of 1.5 mm/s. In Fig. 3, the driven needle trajectories are shown in the hexapod coordinate system. The insertions are performed at different starting positions relative to the US volume by varying the needle height $z_\mathrm{{H}}$ ([−8, −10, −13, −18] mm) and horizontal $x_\mathrm{{H}}$ position ([−15, −20, −25] mm). In total, our data set with the needle aligned parallel to the US coordinate system contains about 600 US volumes acquired in water and another 600 volumes in liver tissue.

Second, we investigate the feasibility of tracking the needle tip while the needle axis is tilted, hence not aligned to the US axis. We perform needle insertions with the needle rotated around the $x_\mathrm{{H}}$ axis ($\alpha _{x_\mathrm{{H}}}$) and $z_\mathrm{{H}}$ axis ($\alpha _{z_\mathrm{{H}}}$). We use three different needle angles ($\alpha _{z_\mathrm{{H}}}=\pm 5^{\circ }$, $\alpha _{x_\mathrm{{H}}}=-5^{\circ }$) in water. For each needle angle we perform nine insertions with a velocity of 1 mm/s in needle axis direction with different $x_\mathrm{{H}}$ and $z_\mathrm{{H}}$ positions. Our tilted data set contains about 2000 US volumes acquired in water.

### Deep learning approach

We use a 3D DenseNet architecture to directly predict the 3D needle tip position from a US volume as input. Our network is based on a DenseNet-121 architecture [7] while we extend the data processing to three dimensions. For efficient training, we crop the US volume to a FOV of 11.25–40 mm along depth direction and 7.5–32.85 mm in $x_{\mathrm{{US}}}$- and $y_{\mathrm{{US}}}$-direction. Please note that we only crop the US volumes and do not perform any additional pre-processing. We define a regression problem to receive the three-dimensional position vector of the needle tip as the output of the network. All US volumes are fed into the network with labels describing the 3D position of the needle tip. We distinguish between experiments where we use the recorded needle tip positions in hexapod coordinates ($\text {COS}_\text {H}$) as the training target and the positions in US coordinates ($\text {COS}_\text {US}$) as the training target. For our quantitative analysis we use the hexapod coordinates as training target as they are more precise and eliminate additional inaccuracies due to US image distortions in the calibration between ultrasound and hexapod. All networks are trained for 800 epochs with a batch size of 8, a learning rate of ${1 \times {10^{-3}}}$, using the Adam optimizer [8]. We define the loss function as the mean squared error between the label and the predicted vector of the needle position. For testing, we use the model which shows best performance on the validation data set.

For our experiments with the needle axis parallel to the US coordinate axis, we train two individual networks on US data acquired either in water or from the liver phantom. For each network, we perform a fivefold cross-validation on the twelve insertion data sets acquired. For each fold, we define two insertion paths for testing, two insertion paths for validation, and the remaining ones for training. The test data set remains the same for all five folds. For the test data, we use an insertion path positioned at a medium depth and one positioned at a lower depth of the US volume, hereafter referred to as the upper and bottom test set, respectively. For the validation data set, we use a new pair of insertion paths for each fold. We make sure these two paths do not lie in the same plane along the $z_{\text {H}}$-axis or $x_{\text {H}}$-axis. Figure 3 shows the respective data split for fold 1.

For the data set with tilted needle axis, we perform a fivefold cross-validation on the nine insertion data sets acquired. For each fold, we randomly define three insertion paths for testing (one path per needle angle), six for validation (two paths per needle angle), and the remaining ones for training.

The trainings are performed on a NVIDIA GeForce RTX 4090 GPU.

### Conventional needle tip detection

**Fig. 4:**
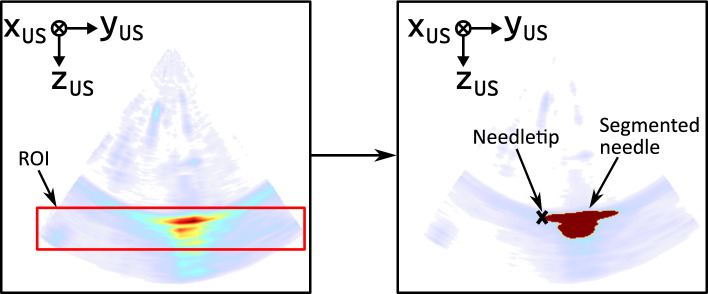
Conventional needle tip tracking: main steps of the conventional segmentation method for needle tip tracking. First, a ROI is defined. Second, the needle is segmented and the needle tip is computed. The volumes are visualized using maximum intensity projection and the jet colormap

The main steps of the conventional segmentation approach (CSM) for needle tip detection are shown in Fig. 4. First, we identify the region of interest (ROI) containing the needle. For this, we assume that the needle is the largest structure in the US image. We apply a median filter (size=[25, 3, 3]) and search for the largest contiguous area with pixel values greater than 128 in the US image. We define a ROI that is centered in the largest structure with ± 25 pixels in the $x_\text {US}$- and $z_\text {US}$-direction. The $y_\text {US}$-direction is not cropped. In the next step, the needle is segmented in the original US image, cropped to the ROI and its needle tip is detected. We perform a binary image segmentation based on a fast marching method using weights based on weighted grayscale differences. We define the needle tip as the farthest point from the edge of the segmented structure.

### Experiments and metrics

In our conducted experiments, we mainly differ between the chosen method for needle tip detection and the medium in which we perform the insertions. First, we evaluate the tracking performance of our conventional segmentation approach compared to our deep learning approach. For this analysis, we use the parallel needle insertion data in water only. Second, we compare the needle tip prediction performance when using the hexapod position as training label compared to using training labels in the US coordinate system. Third we evaluate the performance on a liver data set. In the end, we evaluate the performance on a data set with tilted needle orientations. We evaluate the needle tracking performance of the different experiments based on the mean absolute translation error

1 $$\begin{aligned} e(x_\mathrm{{H}}, y_\mathrm{{H}}, z_\mathrm{{H}}) = \sum _{i=1}^{N}|l_i-p_i| \end{aligned}$$

over *N* US volumes with $l_i$ denoting the training label and $p_i$ the predicted target position.

## Results

Table 1 shows the needle tip tracking errors. The conventional needle tip tracking results in a mean absolute translation error of 1.32 ± 0.73 mm. In comparison, our deep learning approach outperforms the conventional method. Using the water data set with hexapod as training target results in a mean translation error of only 0.54 ± 0.15 mm which is an error reduction by nearly 1 mm. When comparing the different training targets (hexapod- and US coordinate system), similar errors occur. Using the data set with tilted needle orientations ($\alpha _\mathrm{{N}}$), we report a slightly decreased error of 0.17 mm. Our DenseNet-121 leads to an inference time for processing a 3D US volume of 0.01 s.

In addition to the mean position errors, Table 1 shows the individual position errors along the hexapod coordinate axis. For the conventional segmentation approach, particularly large errors are shown along the $x_\text {H}$ axis.

Our experiments on chicken liver tissue demonstrate the ex vivo applicability of our deep learning method for needle tip tracking. However, an increase in tracking errors can be observed along all axes compared to our experiments in water.

**Fig. 5:**
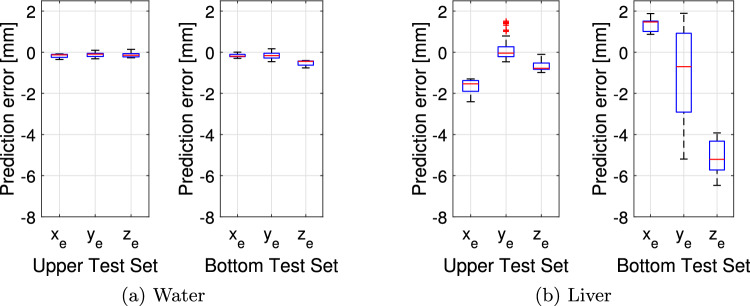
Boxplots showing the needle tip prediction error for the trainings data set with hexapod position as training targets ($\text {COS}_\text {H}$). The error is averaged over all folds. It is differed between the upper test set and bottom test set

In Fig. 5, the mean position errors for the test insertion paths acquired in the upper and lower part of the US volume are analyzed separately. While similarly good position estimates are obtained in water for insertion at both imaging depths, large deviations are observed for liver tissue in the case of insertion in the lower part of the US volume. In particular, there are deviations of up to 7.09 mm for the estimates along the $z_\text {H}$ axes. However, in the case of the upper test path, the needle tip positions still can be predicted with sub-millimeter accuracy.

**Table 1:** Mean absolute error and standard deviation of the needle tip position in water as well as in liver

Method	Target	Medium	e	$e_{x_\text {H}}$	$e_{y_\text {H}}$	$e_{z_\text {H}}$
CSM	$\text {COS}_\text {H}$	Water	1.32 ± 0.73	1.48 ± 0.83	1.79 ± 2.10	0.70 ± 0.90
DenseNet	$\text {COS}_\text {H}$	Water	0.54 ± 0.15	0.79 ± 0.49	0.37 ± 0.24	0.45 ± 0.31
DenseNet	$\text {COS}_\text {US}$	Water	0.48 ± 0.62	0.62 ± 0.38	0.22 ± 0.17	0.61 ± 0.38
CSM	$\text {COS}_\text {H}$	Liver	2.21 ± 1.05	1.77 ± 0.55	3.89 ± 3.07	0.99 ± 0.91
DenseNet	$\text {COS}_\text {H}$	Liver	1.54 ± 0.73	1.60 ± 1.36	0.96 ± 1.43	2.05 ± 1.98
DenseNet	$\text {COS}_\text {H}$	Water; $\alpha _{\mathrm{{N}}}$	0.37 ± 0.19	0.43 ± 0.41	0.26 ± 0.25	0.40 ± 0.43

The mean error *e* across all coordinate axes is shown as well as the errors along $x_\text {H}$, $y_\text {H}$ and $z_\text {H}$. It is differed between the conventional segmentation method (CSM) and the DenseNet approach. $\alpha _{N}$ indicates the data set with tilted needle orientations

## Discussion

The results presented in this work demonstrate the potential of using low-resolution US volumes for needle tracking in real time by deploying a deep learning algorithm. We achieve a high volume acquisition frequency of 4 Hz and report an inference time of 0.01 s for processing. While training targets in ultrasound coordinates are closer to the clinical use case, our study has demonstrated that similar errors occur in hexapod coordinates. This validates the use of hexapod coordinates in our approach, as error rates between the two coordinate systems are comparable. When analyzing the position errors axis-wise, our deep learning approach performs comparatively well on all axes for experiments in water while the CSM algorithm reflects substantial deviations. Keeping the almost parallel orientation of the hexapod and US coordinate axes in mind, errors in $x_\text {H}$ and $y_\text {H}$ as well as $z_\text {H}$ can be interpreted as deviations in estimating the needle tip in the axial plane or depth of the US volume, respectively. Using the deep learning approach a decrease in errors can be noted when using the tilted data set as it contains a larger variety of needle tip positions. For the insertions in chicken liver tissue, a decrease in accuracy can be observed for both approaches. However, the errors for the CSM algorithm are again substantially higher. In particular, the estimation of the $y_\text {H}$ position becomes inaccurate, potentially revealing a wrong estimation of the needle tip along the needle shaft. For our deep learning approach, there are noticeable deviations in the estimation of the $z_\text {H}$-position. When considering these results separately for the insertion depths (Fig. 5), the deviations particularly correspond to the insertion in the lower part of the US volume. This might be related to additional imaging artifacts as the liver tissue contains more structures and boundaries than water which makes needle tip detection more difficult. Adding more training data with a higher variety in $z_\text {H}$-positions could help to reduce this influence. However, our accuracy of needle tip prediction achieved in liver tissue is comparable to those in the current literature where high-resolution US images were used [10, 12].

Looking at our acquired US volumes, detecting the needle tip or at least the needle shaft is rather difficult. Consequently, the accuracy of the conventional segmentation approach is also not particularly high. On the other hand, our trained network seems to recognize decisive patterns in the image data that enable precise regression to a 3D needle tip position. Our quantitative results for needle insertion in water and chicken liver tissue underline these statements. In summary, we demonstrate that our network is capable of predicting the 3D needle position from sparsely sampled US volumes to enable real time needle tracking.

## Conclusion

We present a deep learning approach for detecting needle tips aligned parallel to the US coordinate axis as well as tilted needle tips in low-resolution US volumes acquired with a 16 $\times$ 16 matrix transducer. Our approach enables 3D needle tip position estimation with an accuracy of 0.37±0.19 mm in water and 1.54±0.73 mm in liver tissue. These results are comparable to those in current literature which use high-resolution US volumes. In future work, more complex network structures could be investigated to reach even better predictions in tissue structures. Furthermore, additional training data with more variety in depth positions could increase the needle tip prediction performance. The real-time capability of 3D needle tip detections offers new possibilities for needle insertions. For example, it enables the use of 3D needle tracking in robotic medical interventions to facilitate and improve real time navigation.
